# Limited Interaction Targeted Epidemiology of HIV in Sexual and Gender Minority American Adolescents and Adults: Feasibility of the Keeping it LITE Study

**DOI:** 10.2196/30761

**Published:** 2021-11-26

**Authors:** Neil Gleason, Pedro A Serrano, Alejandro Muñoz, Audrey French, Sybil Hosek

**Affiliations:** 1 University of Washington Seattle, WA United States; 2 Ruth M Rothstein CORE Center Cook County Health Chicago, IL United States; 3 Division of Infectious Disease Stroger Hospital Chicago, IL United States; 4 Department of Psychiatry Stroger Hospital Chicago, IL United States

**Keywords:** social epidemiology, adolescents and young adults, sexual and gender minorities, HIV testing

## Abstract

**Background:**

HIV infection rates among sexual minority men and transgender individuals, particularly adolescents and young adults, remain elevated in the United States despite continued improvement in the HIV public health response. However, there remains a knowledge gap in understanding the barriers faced by this community in receiving HIV care and prevention resources. To address this, the Keeping it LITE study was conducted to assess HIV risk factors and barriers to preventive treatment in a large national cohort of young sexual minority men and transgender individuals at high risk of HIV infection.

**Objective:**

This study aims to evaluate the feasibility of enrolling a large remote cohort, challenges encountered in recruitment, and adjustments made to address these challenges.

**Methods:**

A large national cohort (n=3444) of young sexual minority men and transgender individuals were recruited. Participants were recruited via advertisements on social media; social apps for lesbian, gay, bisexual, transgender, and queer individuals; print advertising; and word-of-mouth. Before enrolling, participants verified their HIV status using an at-home HIV test or by providing their own testing documentation. Descriptive statistics were generated, and a series of logistic regressions were conducted to evaluate demographic differences between recruitment methods, HIV testing methods, and enrollment status.

**Results:**

The Keeping it LITE study was particularly successful in recruiting participants via social media, with over half of the participants recruited from advertisements on social media platforms such as Facebook, Instagram, and Snapchat. Participants were also recruited via word-of-mouth; lesbian, gay, bisexual, transgender, and queer apps (ie, Grindr, Scruff); and print advertisements, and participants recruited from these sources tended to be older and have a higher risk profile. The study was also successful in recruiting a large sample of transgender youth, particularly transgender men and nonbinary individuals. At-home HIV testing was acceptable and more heavily used by younger participants, although several barriers were encountered and overcome in the implementation of this testing. The study had more limited success in recruiting participants aged 13-17 years because of lower enrollment rates and barriers to advertising on social media platforms. The implications of these findings for the future development of HIV research and intervention protocols among sexual minorities and trans youth are discussed.

**Conclusions:**

The methods used in the Keeping it LITE study, particularly recruitment via social media, were found to be feasible and acceptable to participants.

## Introduction

### Background

Despite advances in HIV diagnostics, care, and prevention strategies, HIV infection rates among adolescent and young adult sexual minority men and transgender persons continue to rise in the United States. In 2018, 69% of HIV infections in the United States were attributed to male-to-male sexual contact, with sexual minority men ages 25-34 reporting the highest rates of new diagnoses [1]. Although there continues to be a paucity of research on HIV among transgender youth, 2 studies of young transgender women identified self-reported rates of HIV between 19%-22%, and recent epidemiological studies have demonstrated that many trans masculine adults who have sex with cisgender men (transsexual minority men) may have elevated HIV risk [2,3]. In addition, whereas men who have sex with both men and women (eg, bisexual men and straight-identified men who have sex with men) are less likely to be HIV positive than gay men, they are still more than five times as likely to be HIV positive than men who have sex exclusively with women [4]. Although rates of HIV infection have decreased among many risk groups over the past few years, HIV infection continues to disproportionately impact Black and Hispanic or Latinx sexual minority men and transgender persons, and the most striking racial disparities are found among the youngest sexual minority men [1].

Adolescents and young adults are at an increased risk of HIV infection because of biological, cognitive, psychological, and social changes that occur during this distinct developmental phase [5,6]. Adolescence and emerging adulthood can span the late teens through the late twenties and is characterized by change, instability, and an exploration of love, work, and worldviews [7,8]. Multiple co-occurring vulnerabilities, such as lack of power and resources, exacerbate HIV risk during this time, and the vulnerability of youth who are exploring their sexual orientation or coming to terms with their gender identity can be compounded by stigma and rejection from peers, caregivers, and institutions [9]. These vulnerabilities are exacerbated by coepidemics of poverty and violence as well as the syndemics of mental health issues, drug abuse, and victimization. Despite local and national initiatives, including widespread testing, treatment as prevention, educational programming, and the availability of pre-exposure prophylaxis (PrEP) and postexposure prophylaxis (PEP), new HIV infections among young sexual minority men and transgender persons persist. In fact, while the number of HIV infections in sexual minority men and transgender persons between 2014 and 2018 decreased among most age groups, annual increases were seen among those aged 25-34 years [1].

Unfortunately, young and very young sexual minority men and transgender persons are less likely to be aware of and avail themselves of HIV testing and effective prevention technologies because of barriers at multiple levels. A significant knowledge gap exists in understanding the role of these barriers in the ongoing epidemic of HIV acquisition among young sexual minority men and transgender persons. Therefore, there is an urgent need to articulate the epidemiology of HIV acquisition in this population to target culturally and age-appropriate prevention interventions toward those at the highest risk of infection.

### Objectives

To address this knowledge gap, the *Keeping it LITE: Exploring HIV Risk in Vulnerable Youth with Limited Interaction* (UG3 AI 133669 & UH3 AI 133676) study was designed to assess HIV risk factors and barriers to preventive treatment in a large national cohort of youth and young adults at high risk of HIV infection. The purpose of this study is to evaluate the feasibility of enrolling (via web-based recruitment) a large, remote cohort of diverse sexual minority men and transgender persons aged 13-34 years (including 20% ages 13-17 years) at high risk of HIV infection and discuss challenges encountered in recruitment and enrollment, and adjustments made to address these challenges. Specifically, we will (1) examine which recruitment methods were most successful for specific demographics; (2) examine the characteristics of individuals who screened eligible for the cohort but did not enroll to identify barriers to enrollment; and (3) evaluate the feasibility of using HIV self-testing as a study procedure.

## Methods

### Study Development

#### Youth Advisory Board

During the study development phase, a youth advisory board (YAB) was organized to provide insight into methods of recruitment and retention for young sexual minority men and transgender persons. The YAB consisted of 18 lesbian, gay, bisexual, transgender, and queer (LGBTQ+) youths of color aged 13-24 years. These individuals were recruited via existing relationships with clinical trial networks and from participants enrolled in Keeping it LITE. YAB members reviewed community engagement, outreach, education, recruitment, retention, data collection, incentive, and linkage to care plans and provided feedback.

#### Recruitment Materials

**Figure 1:**
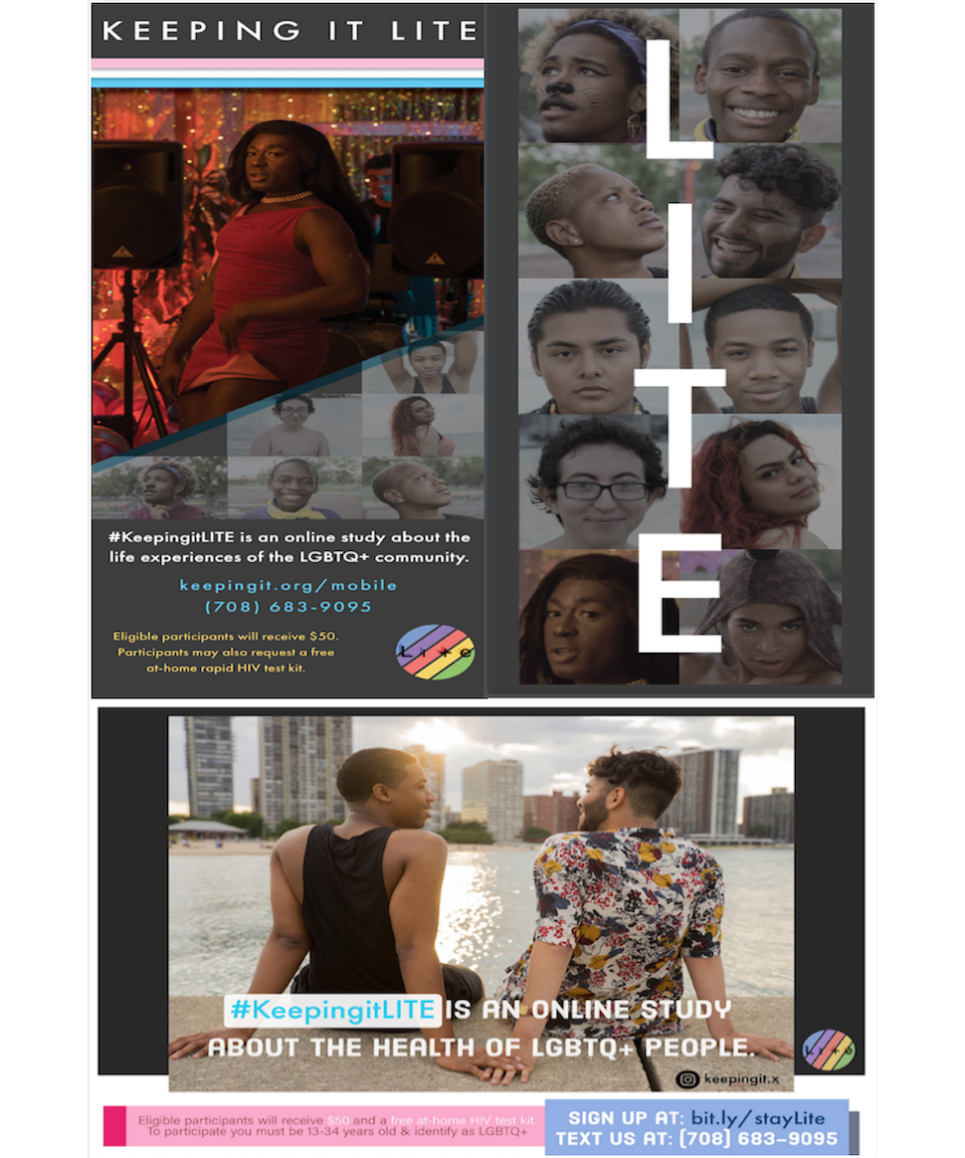
Examples of advertisements developed for this study.

Advertising and marketing media for the study were developed in collaboration with graphic designers and the YAB. At each stage of development, drafts of recruitment materials were shown to the YAB to assess whether they were understandable, culturally appropriate, and engaging. Examples of feedback from the YAB include suggestions to emphasize visuals over text, to use colors from the LGBTQ pride and trans pride flags to make messaging pop out, to use the same account name on the study website and social media accounts, and to post frequently on social media. The marketing and branding strategy was carefully designed to include all gender and racial or ethnic identities. Figure 1 shows examples of advertisements developed for this study.

### Recruitment Procedures

Recruitment advertisements were distributed over social media platforms and mobile apps viewable on all internet-enabled smart devices with specified regions, demographics, and times. The YAB was consulted about methods of recruitment via LGBTQ-specific social media, and they identified Grindr, Jack’d, Scruff, and Adam4Adam as the apps most commonly used by young sexual minority men or transgender persons. Advertisements were placed on Facebook, Instagram, Snapchat, Spotify, Twitter, YouTube, Grindr, Hornet, Growlr, and Scruff. Print advertisements were also placed in Chicago-area public transit systems and distributed at a limited number of in-person events in Chicago. Finally, participants were also recruited by word-of-mouth, with current participants in the study referring others to join. Participants were given an incentive (US $20) for each of their enrolled referrals. Recruitment was originally limited to the Chicago area but later expanded to cities across the United States.

### Barriers in Social Media Recruitment

Before launching recruitment, Facebook and Instagram removed the ability to target advertisements through sexual orientation identity [10]. Advertisement targeting was therefore switched from using the built-in sexual orientation indicator to a mix of key ad words to target LGBTQ+ youth and adults (such as media or consumer goods that were popular among this demographic based on suggestions from YAB). Snapchat also changed its advertising policy to block minors from receiving advertisements for clinical trials or research studies. Thus, this medium could only be used to recruit participants aged ≥18 years. Finally, some study advertisements were flagged as inappropriate content for minors on Facebook and Instagram. These advertisements typically featured cisgender, transgender, and nonbinary youth holding hands, hugging, giving each other endearing looks, etc. It was noted that photos featuring individuals in summer scenes (eg, beach, swimwear, and shorts) were most likely to be flagged. These advertisements could continue to be used in posts by the study’s social media accounts on Facebook and Instagram but were not able to be used in paid advertising.

### Prescreening Questionnaire

Study advertisements directed potential participants to a prescreening questionnaire with further information about the study and a series of questions to assess eligibility. To enroll in the study, participants were required to be located in the United States, identify as a cis- or transgender man or transgender woman, or nonbinary person who has sex with persons assigned male at birth, between the ages of 13 and 34 years, report being HIV negative or receiving an HIV diagnosis in the previous year, and report at least one of the following in the last 6 months: (1) condomless anal sex; (2) sex with an HIV-positive partner; or (3) a bacterial sexually transmitted infection (STI). Participants aged <18 years were enrolled as allowable in their state of residence (6 states do not allow minors to access HIV or STI testing services independently until the ages of 14-16) [11].

In the course of recruitment, it was found that many potential participants under the age of 18 did not meet eligibility criteria because they did not report sufficient sexual risk (ie, reporting no anal sex, HIV-positive partners, or STIs). To facilitate recruitment of the age group, criteria were adjusted to allow participants under 18 to be eligible if they reported engaging in unprotected oral sex with a person assigned male at birth.

### Enrollment Procedures

Potential participants who met the inclusion criteria were directed to an electronic consent form to read and sign. Only one enrollment was allowed per IP address to ensure that individuals were enrolled only once. In addition, participants agreed to disclose their name, date of birth, street address, phone number, and email through a secure, Health Insurance Portability and Accountability Act–compliant system. This information was reviewed by the study staff to ensure that the participant was not previously enrolled before a unique participant ID number was assigned.

### Assessments

Once informed consent was obtained, the participants completed the HIV status verification. Participants could choose to have an at-home HIV test kit mailed to them or by securely uploading an HIV health record, such as an HIV test lab report, antiretroviral treatment, or PrEP prescription obtained within the last 3 months. Participants then completed a 30-minute baseline assessment with questions assessing demographics, social determinants of health (health insurance status, use of public assistance, immigration status, employment, housing, education, etc), health care experiences, substance use, mental health (social support, attitudes, stigma, discrimination, depression, posttraumatic stress disorder), sexual risk behavior, and HIV prevention engagement. The baseline assessment also included an optional question asking participants how they heard about the study (word-of-mouth, print advertisement, social media, or LGBTQ+ app). The participants completed follow-up evaluations every 6 months. For each follow-up evaluation, they received an automated email prompting them to complete a questionnaire and repeat their HIV testing or status verification.

### Retention Strategies

When participants entered their follow-up window (180 days from the last visit), automated email reminders were sent at 1, 3, 7, 14, and 28 days. Participants were messaged semiannually even if they missed one or more previous interactions. On holidays or special awareness days, such as World AIDS Day and National Youth HIV & AIDS Awareness Day, participants were encouraged to participate in contests posted on social media to win small gifts through lotteries.

### Measures

#### HIV Testing

HIV testing was conducted using oral specimen HIV self-testing kits. This testing method was chosen after consultation with the YAB. The YAB expressed a preference for oral specimens over a self-collected blood specimen, and for the ability to upload results via an encrypted web portal over mailing a test back to the researchers. The study staff purchased large volumes of test kits and shipped them to the participants within a week of their request date. The packages included instructions for self-administration in easy-to-understand language and diagrams as well as a Centers for Disease Control and Prevention self-testing educational pamphlet. Participants were instructed to take a picture of the test results and upload it to the encrypted study survey platform. This platform also included links to video tutorials, demonstrating the self-testing procedure. Several ways to securely reach the staff were prominent in the testing instructions with specific instructions should the test be reactive. Participants who experienced challenges receiving their tests by mail received one-on-one troubleshooting with the study staff. This testing method proved inexpensive, with the total cost of the test, packaging, and postage totaling less than US $20.

To ensure participant privacy and confidentiality with regard to the testing kits, mail packaging was as discreet as possible, and a fully encrypted, secure, Health Insurance Portability and Accountability Act–compliant web-based data collection program was used to send results. Participants were informed about privacy measures in place for their test data through the informed consent process, and throughout the testing process, participants were encouraged to express feedback or concerns through confidential communication methods. To ensure the privacy and safety of sexual and gender minority participants, we sought and were granted a waiver of the requirement of parental consent for participation.

#### Adjunct Surveys

Three adjunct surveys were added to the study to evaluate the impact of relevant current events. This included a brief survey in late November 2019-December 2019 to evaluate the effects of misleading advertisements seeking plaintiffs in a lawsuit against Gilead for Truvada toxicities [12]; a survey in May and September of 2020 and May of 2021 to ascertain how the COVID-19 pandemic was affecting the mental, financial, and sexual health of participants [13]; and a short survey in October of 2020 to better understand youth aged 13-17 years who screened eligible but chose not to enroll. The results of the first two surveys are beyond the scope of this study and have been presented and discussed elsewhere [12,13]. The methods of the latter survey are discussed here to provide insight into the recruitment and retention of minor participants.

In October 2020, 246 participants who had consented to participate between December 2017 and December 2019 were between the ages of 13 and 17 at the time of consent but failed to complete baseline HIV testing (which would have completed study enrollment) were invited to participate in a short survey. The survey included practical questions about the mechanics of receiving an HIV test, privacy concerns, data use, electronic incentives, as well as issues of stigma, disclosure, risk perception, and fear of test results.

### Participant Compensation

Participants were compensated US $50 per study visit after the HIV test result or status verification was received, and the survey was completed. Participants received compensation through a web-based gift card distribution system that allowed them to choose between a variety of electronic gift cards or a prepaid debit card. The choice to use this web-based system was made after receiving feedback from participants that they wanted to receive their payments as quickly as possible and after consultation with the YAB. For the ad hoc surveys, the study team experimented with random lottery and flat-rate compensation, but ultimately found no difference in response rates. Participants were also compensated with US $20 for completing adjunct surveys.

### Statistical Analysis

Descriptive statistics were generated for the data, including frequencies, means, SDs, and odds ratios (ORs) for participant demographics and recruitment methods, testing methods, and enrollment status. A series of logistic regressions were conducted to evaluate differences in participant demographics between recruitment methods, HIV testing methods, and enrollment status. Analyses were performed using JASP version 0.14.1 [14].

## Results

### Sample Characteristics

**Figure 2:**
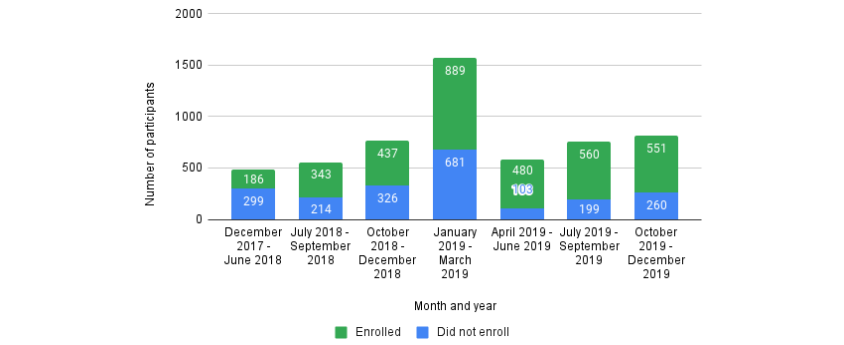
Enrollment activity by study quarter.

**Figure 3:**
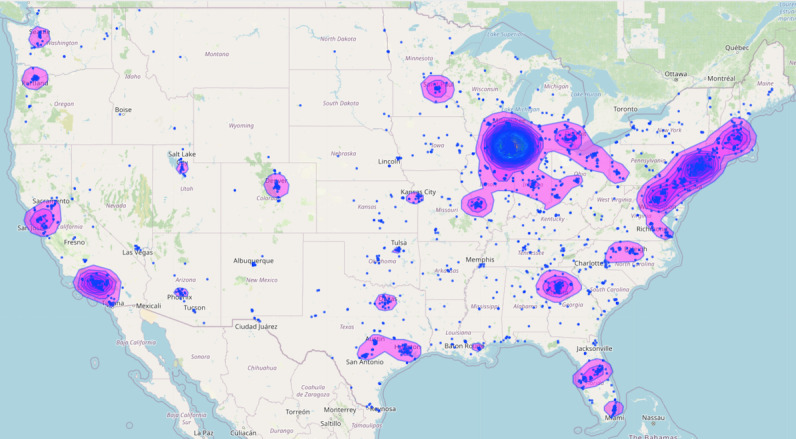
Map of participant enrollment by location.

The demographic characteristics of the enrolled participants are shown in Table 1. Participants were recruited from December 2017 to December 2019 (Figure 2) and recruited from across the United States (Figure 3). Most participants identified as cisgender men (2613/3444, 75.87%), with most cisgender men in the study identifying as gay (2089/2613, 97.94%). Furthermore, 1.91% (66/3444) of participants identified as transgender women, 8.04% (277/3444) identified as transgender men, and 14.16% (488/3444) of participants identified as genderqueer or gender nonconforming. Nearly half of the participants were identified as persons of color (1631/3444, 47.35%).

**Table 1:** Characteristics of enrolled participants (N=3444).

Characteristics				Total (N=3444)		White (n=1813)		Latinx (n=680)		Black (n=417)		Asian or Pacific Islander (n=154)		Other (n=380)
Age (years), mean (SD)				24.6 (4.8)		25.0 (4.8)		24.2 (4.9)		24.4 (4.7)		24.6 (4.8)		23.7 (4.8)
**Sexual orientation and gender, n (%)**														
	**Cisgender men**													
		Gay	2098 (60.91)		1165 (64.25)		416 (61.18)		227 (54.43)		100 (64.94)		190 (50)	
		Other	506 (14.69)		201 (11.09)		119 (17.5)		88 (21.1)		25 (16.23)		73 (19.21)	
		Straight	9 (0.26)		4 (0.22)		1 (0.15)		2 (0.48)		0 (0)		2 (0.53)	
	**Nonbinary**													
		Gay	138 (4)		54 (2.97)		29 (4.26)		34 (8.15)		4 (2.59)		17 (4.47)	
		Other	345 (10.02)		184 (10.15)		56 (8.23)		43 (10.31)		15 (9.74)		47 (12.37)	
		Straight	5 (0.14)		0 (0)		1 (0.15)		2 (0.48)		0 (0)		2 (0.53)	
	**Trans men**													
		Gay	54 (1.57)		36 (1.99)		7 (1.03)		1 (0.24)		2 (1.29)		8 (2.11)	
		Other	213 (6.18)		129 (7.12)		33 (4.85)		10 (2.39)		8 (5.19)		33 (8.68)	
		Straight	10 (0.29)		6 (0.33)		3 (0.44)		0 (0)		0 (0)		1 (0.26)	
	**Trans women**													
		Gay	12 (0.35)		6 (0.33)		2 (0.29)		3 (0.72)		0 (0)		1 (0.26)	
		Other	44 (1.28)		28 (1.54)		7 (1.03)		5 (1.19)		0 (0)		4 (1.05)	
		Straight	10 (0.29)		0 (0)		6 (0.88)		2 (0.48)		0 (0)		2 (0.5)	
**Location^a^, n (%)**														
	**EHE2^b^**													
		Midwest	1124 (32.64)		526 (29.01)		237 (34.85)		200 (487.96)		51 (33.12)		110 (28.95)	
		South	449 (13.04)		226 (12.46)		80 (11.76)		82 (19.66)		13 (8.44)		48 (12.63)	
		Northeast	183 (5.31)		89 (4.91)		35 (5.15)		18 (4.32)		18 (11.69)		23 (6.05)	
		West	241 (6.99)		106 (5.85)		76 (11.18)		14 (3.36)		19 (12.34)		26 (6.84)	
	**Non-EHE**													
		Midwest	572 (16.61)		371 (20.46)		75 (11.03)		35 (8.39)		20 (12.98)		71 (10.44)	
		South	358 (10.39)		195 (10.76)		73 (10.74)		45 (10.79)		8 (5.19)		37 (9.74)	
		Northeast	291 (8.45)		174 (9.59)		50 (7.35)		19 (4.56)		12 (7.79)		36 (9.47)	
		West	226 (6.56)		126 (6.95)		54 (7.94)		4 (0.96)		13 (8.44)		29 (7.63)	
**HIV status, n (%)**														
	HIV negative			2561 (74.36)		1388 (76.56)		507 (74.56)		258 (61.87)		117 (75.97)		291 (76.58)
	HIV negative and on PrEP^c^ or PEP^d^			769 (22.32)		407 (22.45)		151 (22.21)		99 (23.74)		36 (23.38)		76 (20)
	HIV positive			114 (3.31)		18 (0.99)		22 (3.23)		60 (14.39)		1 (0.65)		13 (3.42)
**Permanent housing^a^, n (%)**														
	Yes			3208 (93.15)		1742 (96.08)		618 (90.88)		347 (83.21)		149 (96.75)		352 (92.63)
	No			204 (5.92)		57 (3.14)		55 (8.09)		65 (15.59)		4 (2.59)		23 (6.05)
**Education^a^, n (%)**														
	≤Bachelor’s degree			1548 (44.95)		899 (49.59)		276 (42.59)		141 (33.81)		95 (61.69)		137 (36.05)
	≥Bachelor’s degree			1670 (48.49)		789 (43.52)		372 (54.71)		253 (60.67)		50 (32.48)		206 (54.21)
**Insurance status^a^, n (%)**														
	Insured			2602 (75.56)		1423 (78.49)		501 (73.68)		278 (66.67)		129 (83.77)		271 (71.32)
	Not Insured			334 (9.69)		118 (6.51)		104 (15.29)		66 (15.83)		7 (4.54)		39 (10.26)
**Employment^a^, n (%)**														
	Yes			1937 (56.24)		1098 (60.56)		365 (53.68)		216 (51.79)		74 (48.05)		184 (48.42)
	No			1026 (29.79)		457 (25.21)		236 (34.71)		155 (37.17)		43 (27.92)		135 (35.52)
**Any income^a^, n (%)**														
	Yes			2786 (80.89)		1476 (81.41)		563 (82.79)		322 (77.22)		131 (85.06)		294 (77.37)
	No			436 (12.66)		215 (11.86)		86 (12.65)		72 (17.27)		14 (9.09)		49 (12.89)
**Number of risk criteria^e^, n (%)**														
	2 or more			1241 (36.03)		584 (32.21)		256 (37.65)		208 (49.88)		51 (33.12)		142 (37.37)
	Less than 2			2203 (63.96)		1229 (67.79)		424 (62.35)		209 (50.12)		103 (66.88)		238 (62.63)
**PrEP indicated^f^, n (%)**														
	Yes			2972 (86.29)		1594 (87.92)		599 (88.09)		328 (78.66)		138 (89.61)		313 (82.37)
	No			472 (13.7)		219 (12.08)		81 (11.91)		89 (21.34)		16 (10.39)		67 (17.63)
**Substance use^a,g^, n (%)**														
	Yes			533 (15.48)		276 (15.22)		109 (16.03)		49 (11.75)		21 (13.64)		78 (20.53)
	No			2133 (61.93)		1206 (66.52)		398 (58.53)		238 (57.07)		90 (58.44)		201 (52.89)

^a^Denotes missing data.

^b^EHE: ending the HIV epidemic priority jurisdictions [15].

^c^PrEP: pre-exposure prophylaxis.

^d^PEP: postexposure prophylaxis.

^e^Risk criteria: (1) inconsistent condom use, (2) HIV-positive partner, and (3) bacterial sexually transmitted infection in the past 6 months.

^f^PrEP indication is based on Centers for Disease Control and Prevention’s draft of clinical practice guidelines [16]: HIV negative, recent anal or vaginal sex, and one of the following: inconsistent condom use, HIV-positive partner, or bacterial sexually transmitted infection in past 6 months.

^g^Substance use: cocaine, meth, or heroin use in past 6 months.

### Social Media Engagement and Recruitment Sources

During the study recruitment period, digital advertisements for the study were seen (ie, displayed on someone’s social media feed) over 2 million times and clicked open over 720,000 times. Social media platforms such as Snapchat, Instagram, and Facebook accounted for a majority (about 9 out of 10) of clicks, whereas about 1 in 10 clicks were from dating apps such as Grindr, Hornet, Growlr, and Scruff. The study Facebook page accrued more than 4200 likes, and the study Instagram account accrued more than 2700 followers. The website for the study received over 35,000 unique visitors.

A total of 2649 participants reported how they heard about the study. Descriptive statistics, ORs, and results of the logistic regression analysis are displayed in Table 2. The most commonly reported method of recruitment was social media (1684/2649, 63.57%), although some methods were more effective for certain demographics than others. A series of logistic regressions indicated advertisements on LGBT+ apps such as Grindr more commonly enrolled older participants with a higher risk profile, whereas advertisements on social media apps such as Instagram and Snapchat tended to attract younger participants with lower risk profiles. Cisgender men were more likely than transgender participants to hear about the study from Grindr or other LGBT apps, and participants aged <20 years were more likely to hear about the study from social media than participants ≥20 years. Word-of-mouth and LGBTQ+ app methods also tended to enroll more participants who were HIV positive or using PrEP or PEP. The results of the multivariate model confirmed the independence of these associations (Table 2).

**Table 2:** Participant characteristics by recruitment method (n=2649).

Characteristics				Total		Social media		Word-of-mouth		LGBTQ+^a^ apps		Print ads		Social media versus all other, univariate OR^b^ (95% CI)		*P* value^c^			Multivariate OR (95% CI)	
Total, n (%)				2649 (100)		1684 (63.61)		499 (18.81)		341 (12.91)		125 (4.61)		—^d^		—			—	
**Age (years), mean (SD)**				24.6 (4.8)		24.3 (4.9)		24.8 (4.6)		26.6 (4.5)		23.5 (4.9)		0.96 (0.94-0.97)		<.001			0.97 (0.95-0.98)	
	30-34		483 (18.2)		282 (16.7)		85 (17)		97 (28.4)		19 (15.2)		Reference							
	25-29		860 (32.5)		506 (30.0)		182 (36.5)		141 (41.3)		31 (24.8)		1.02 (0.81-1.28)							
	20-24		858 (32.4)		570 (33.8)		162 (32.5)		77 (22.6)		49 (39.2)		1.41 (1.12-1.78)							
	18-19		243 (9.2)		165 (9.8)		42 (8.4)		22 (6.5)		14 (11.2)		1.51 (1.09-2.09)							
	13-17		205 (7.7)		161 (9.6)		28 (5.6)		4 (1.2)		12 (9.6)		2.61 (1.78-3.81)							
**Race or gender, n (%)**																				
	**Sexual minority men**																<.001			
		White	1091 (41.21)		725 (43.13)		168 (33.68)		154 (45.21)		44 (35.18)		Reference					Reference		
		Latinx	434 (16.43)		276 (16.41)		71 (14.21)		68 (19.89)		19 (15.21)		0.88 (0.70-1.11)					0.82 (0.63-1.06)		
		Black	250 (9.37)		122 (7.18)		68 (13.57)		47 (13.78)		13 (10.45)		0.48 (0.36-0.64)					0.58 (0.42-0.80)		
		Asian	102 (3.89)		69 (4.08)		20 (4.03)		12 (3.54)		1 (0.79)		1.06 (0.68-1.63)					1.06 (0.66-1.69)		
		Other	206 (7.78)		137 (8.12)		37 (7.42)		23 (6.71)		9 (7.21)		1.00 (0.73-1.37)					1.01 (0.72-1.43)		
	**Trans or nonbinary**																			
		White	297 (11.22)		199 (11.77)		66 (13.23)		15 (4.56)		17 (13.61)		1.03 (0.78-1.35)		—			0.89 (0.66-1.20)		
		Latinx	101 (3.81)		54 (3.21)		29 (5.79)		9 (2.61)		9 (7.22)		0.58 (0.38-0.87)		—			0.72 (0.45-1.14)		
		Black	67 (2.45)		35 (2.13)		23 (4.56)		3 (0.87)		6 (4.76)		0.55 (0.34-0.91)		—			0.90 (0.52-1.57)		
		Asian	20 (0.82)		12 (0.65)		2 (0.36)		6 (1.81)		0 (0)		0.76 (0.31-1.87)		—			0.85 (0.31-2.33)		
		Other	81 (3.13)		55 (3.33)		15 (3.02)		4 (1.17)		7 (5.61)		1.07 (0.66-1.73)		—			0.85 (0.50-1.45)		
**Location^e^, n (%)**																				
	**EHE^f^**																<.001			
		Midwest	735 (27.71)		252 (15.04)		283 (56.74)		96 (28.19)		104 (83.21)		Reference					Reference		
		South	384 (14.51)		291 (17.34)		43 (8.62)		45 (13.16)		5 (4.02)		6.00 (4.54-7.93)					5.96 (4.48-7.92)		
		Northeast	150 (5.66)		110 (6.47)		18 (3.61)		19 (5.62)		3 (2.37)		5.27 (3.56-7.81)					5.54 (3.71-8.26)		
		West	209 (7.92)		154 (9.12)		16 (3.18)		38 (11.11)		1 (0.77)		5.37 (3.81-7.57)					5.19 (3.66-7.37)		
	**Non-EHE**																			
		Midwest	429 (16.21)		295 (17.53)		68 (13.62)		60 (17.61)		6 (4.78)		4.22 (3.27-5.44)		—			3.54 (2.72-4.60)		
		South	315 (11.87)		245 (14.45)		36 (7.21)		33 (9.72)		1 (0.76)		6.71 (4.94-9.11)		—			6.14 (4.50-8.39)		
		Northeast	232 (8.81)		185 (11.02)		19 (3.76)		24 (7.02)		4 (3.21)		7.54 (5.29-10.75)		—			6.56 (4.58-9.41)		
		West	195 (7.41)		152 (9.02)		16 (3.21)		26 (7.63)		1 (0.78)		6.78 (4.67-9.82)		—			6.14 (4.20-8.96)		
**HIV status, n (%)**																				
	HIV negative		1969 (74.3)		1321 (78.42)		332 (66.45)		221 (64.78)		95 (76.02)		Reference		<.001			Reference		
	PrEP^g^ or PEP^h^		586 (22.1)		320 (19.05)		144 (28.9)		99 (29.0)		23 (18.34)		0.59 (0.49-0.71)					0.78 (0.63-0.97)		
	HIV positive		94 (3.45)		43 (2.63)		23 (4.6)		21 (6.2)		7 (5.6)		0.41 (0.27-0.63)					0.66 (0.40-1.06)		
**Number of risk criteria^i^, n (%)**																				
	2 or more		989 (37.31)		558 (33.08)		234 (46.89)		160 (46.91)		37 (29.56)		Reference		<.001			Reference		
	Less than 2		1660 (62.72)		1126 (66.91)		265 (53.13)		181 (53.12)		88 (70.34)		1.63 (1.38-1.92)		—			1.19 (0.98-1.45)		
**PrEP indicated^j^, n (%)**																				
	Yes		2371 (89.45)		1518 (90.1)		440 (88.21)		315 (92.42)		98 (78.42)		Reference		.16			—		
	No		278 (10.51)		166 (9.88)		59 (11.83)		26 (7.58)		27 (21.57)		0.83 (0.65-1.07)		—			—		
**Substance use^e,k^, n (%)**																				
	Yes		416 (20.56)		253 (15.02)		81 (20.71)		62 (24.17)		20 (20.41)		Reference		.32			—		
	No		1599 (79.34)		1015 (60.27)		311 (79.32)		195 (75.87)		78 (79.58)		1.12 (0.90-1.40)		—			—		

^a^LGBTQ+: lesbian, gay, bisexual, transgender, and queer.

^b^OR: odds ratio.

^c^Results of logistic regression (outcome variable=recruited via social media vs recruited via other method).

^d^Not available.

^e^Denotes missing data.

^f^EHE: ending the HIV epidemic priority jurisdictions [15].

^g^PrEP: pre-exposure prophylaxis.

^h^PEP: postexposure prophylaxis.

^i^Risk criteria: (1) inconsistent condom use, (2) HIV-positive partner, and/or (3) bacterial sexually transmitted infection in the past 6 months.

^j^Pre-exposure prophylaxis indication is based on Centers for Disease Control and Prevention’s draft of clinical practice guidelines [16]: HIV negative, recent anal or vaginal sex, and one of the following: inconsistent condom use, HIV-positive partner, or bacterial sexually transmitted infection in past 6 months.

^k^Substance use: cocaine, meth, or heroin use in past 6 months ≥2 risk criteria: (1) inconsistent condom use, (2) HIV-positive partner, and/or (3**)** bacterial sexually transmitted infection in the past 6 months.

### Qualification and Enrollment

Table 3 displays the enrollment status of all 11,821 participants who completed eligibility screenings. A series of logistic regressions indicated that younger eligible participants were less likely than older eligible participants to enroll in the study. Participants aged 13-17 years were significantly less likely to enroll compared with participants aged 30-34 years (OR 0.44, 95% CI 0.35-0.56). In addition, compared with eligible White cisgender male-identified participants, Asian (OR 0.88, 95% CI 0.62-1.13), Black (OR 0.70, 95% CI 0.58-0.85), and Latinx (OR 0.75, 95% CI 0.64-0.89) participants were less likely to enroll, and transgender and nonbinary individuals were less likely to enroll than cisgender participants. Eligible HIV-positive participants were 1.67 (95% CI 1.20-2.33) times more likely and HIV negative participants on PrEP or PEP were 15.73 (95% CI 11.3-21.9) times more likely to enroll than HIV negative participants not on PrEP or PEP. The results of the multivariate model confirmed the independence of these associations (Table 3).

**Table 3:** Characteristics of participants by enrollment status (n=11,821).

Characteristics					Total		Did not qualify		Did not enroll		Enrolled		Enrolled versus did not enroll, univariate OR^a^ (95% CI)		*P* value^b^			Multivariate OR (95% CI)	
Total, n (%)					11,821 (100)		6295 (53.25)		2082 (17.62)		3444 (29.13)		—^c^		—			—	
**Age (years), mean (SD)**					25.1 (8.1)		25.9 (10.0)		23.6 (4.9)		24.6 (4.8)		1.04 (1.03-1.06)		<.001			1.02 (1.00-1.03)	
	>34			778 (6.6)		778 (12.4)		0 (0)		0 (0)		—							
	30-34			1593 (13.5)		681 (10.8)		293 (14.1)		619 (18)		Reference							
	25-29			2792 (23.6)		1181 (18.8)		546 (26.2)		1065 (30.9)		0.92 (0.78-1.1)							
	20-24			4017 (34.0)		2049 (32.5)		777 (37.3)		1191 (34.6)		0.73 (0.61-0.86)							
	18-19			1386 (11.7)		827 (13.1)		220 (10.6)		339 (9.8)		0.73 (0.59-0.91)							
	13-17			1252 (10.6)		776 (12.3)		246 (11.8)		230 (6.7)		0.44 (0.35-0.56)							
	<13			3 (0)		3 (0)		0 (0)		0 (0)		—							
**Race or gender, n (%)**																			
	**Sexual minority men**															<.001			
		White		3726 (31.45)		1671 (26.48)		685 (32.88)		1370 (39.78)		Reference					Reference		
		Latinx		1405 (11.87)		514 (8.18)		355 (17.09)		536 (15.57)		0.75 (0.64-0.89)					0.79 (0.67-0.94)		
		Black		892 (7.52)		349 (5.48)		226 (10.87)		317 (9.16)		0.70 (0.58-0.85)					0.66 (0.53-0.81)		
		Asian		322 (2.69)		122 (1.89)		75 (3.59)		125 (3.62)		0.83 (0.62-1.13)					0.85 (0.62-1.17)		
		Other		735 (6.16)		295 (4.68)		175 (8.36)		265 (7.66)		0.76 (0.61-0.94)					0.79 (0.63-0.99)		
	**Trans or nonbinary**																		
		White		2476 (20.87)		1773 (28.16)		260 (12.49)		443 (12.89)		0.85 (0.71-1.02)		—			1.12 (0.92-1.36)		
		Latinx		737 (6.19)		473 (7.52)		120 (5.76)		144 (4.19)		0.60 (0.46-0.78)		—			0.71 (0.54-0.94)		
		Black		824 (7.04)		627 (10.02)		97 (4.67)		100 (2.91)		0.52 (0.38-0.69)		—			0.60 (0.44-0.82)		
		Asian		147 (1.23)		108 (1.72)		10 (0.49)		29 (0.77)		1.45 (0.70-2.99)		—			1.85 (0.88-3.85)		
		Other		557 (4.72)		363 (5.77)		79 (3.8)		115 (3.31)		0.73 (0.54-0.98)		—			0.96 (0.70-1.32)		
**Location^d^, n (%)**																			
	**EHE^e^**															.71			—
			Midwest	3666 (31.02)		1893 (30.08)		649 (31.32)		1124 (32.6)		Reference							
			South	1487 (12.57)		769 (12.21)		269 (13)		449 (13.03)		0.96 (0.81-1.15)							
			Northeast	592 (5.02)		300 (4.79)		109 (5.29)		183 (5.31)		0.97 (0.75-1.25)							
			West	772 (6.52)		373 (5.89)		158 (7.62)		241 (6.8)		0.88 (0.70-1.10)							
	**Non-EHE**																		
			Midwest	2431 (20.62)		1486 (23.61)		373 (18.01)		572 (16.56)		0.89 (0.75-1.04)		—			—		
			South	1121 (9.47)		558 (8.86)		205 (9.91)		358 (10.42)		1.01 (0.83-1.23)		—			—		
			Northeast	1011 (8.59)		559 (8.88)		161 (7.81)		291 (8.42)		1.04 (0.84-1.29)		—			—		
			West	730 (6.2)		356 (5.69)		148 (7.12)		226 (6.6)		0.88 (0.70-1.11)		—			—		
**HIV status, n (%)**																			
	HIV negative, not on PrEP^f^ or PEP^g^			10,536 (89.12)		5984 (95.08)		1991 (95.59)		2561 (74.36)		Reference		<.001			Reference		
	HIV negative; on PrEP or PEP			807 (6.82)		0 (0)		38 (1.77)		769 (22.31)		15.73 (11.3-21.9)		—			16.42 (11.7-23.05)		
	HIV positive			478 (4.03)		311 (4.92)		53 (2.53)		114 (3.27)		1.67 (1.20-2.33)		—			2.25 (1.57-3.2)		
**Number of risk criteria^h^, n (%)**																			
	3			649 (5.48)		148 (2.39)		170 (8.22)		331 (9.61)		Reference		<.001			Reference		
	2			1868 (15.77)		539 (8.61)		419 (20.07)		910 (26.38)		1.12 (0.90-1.39)					1.64 (1.28-2.11)		
	1			4905 (41.45)		1876 (29.83)		1186 (57.03)		1843 (53.51)		0.80 (0.65-0.97)					1.55 (1.22-1.96)		
	0			4399 (37.23)		3732 (59.33)		307 (14.67)		360 (10.46)		0.60 (0.47-0.77)					1.4 (1.05-1.88)		

^a^OR: odds ratio.

^b^Results of logistic regression (outcome variable=enrolled vs did not enroll).

^c^Not available.

^d^Denotes missing data.

^e^EHE: ending the HIV epidemic priority jurisdictions [15].

^f^PrEP: pre-exposure prophylaxis.

^g^PEP: postexposure prophylaxis.

^h^Risk criteria: (1) inconsistent condom use, (2) HIV-positive partner, and/or (3) bacterial sexually transmitted infection in the past 6 months.

### HIV Testing Methods

Table 4 displays HIV testing methods used by participants across demographics. A majority of participants (2573/3444, 74.71%) opted to use the at-home HIV testing kits provided by the study. A series of logistic regressions indicated younger participants used the at-home tests at higher rates than older participants, with participants aged 13-17 years being 6.38 (95% CI 6.61-11.25) times more likely to use at-home tests than participants aged 30-34 years. Compared with cisgender White men, cisgender Black men were 0.46 (95% CI 0.36-0.59) times as likely and cisgender Asian men were 0.62 (95% CI 0.42-0.92) times as likely to use at-home tests. Trans and nonbinary participants were more mixed, with White participants having greater odds (OR 1.54, 95% CI 1.17-2.04) and Black participants having lower odds (OR 0.58, 95% CI 0.38-0.90) of using at-home tests. The results of the multivariate model confirmed the independence of these associations except for age and substance use (Table 4).

**Table 4:** Characteristics of participants using HIV self-testing versus other HIV status verification (n=3444).

Characteristics			Total	HIV self-test	Other status verification		Univariate OR^a^ (95% CI)	*P* value^b^			Multivariate OR (95% CI)		
Total, n (%)			3444 (100)	2573 (74.71)	871 (25.29)		—^c^	—			—		
**Age (years), mean (SD)**			24.6 (4.8)	24.3 (4.9)	25.5 (4.4)		0.95 (0.93-0.96)	<.001			1.02 (0.99-1.04)		
	30-34		619 (18.0)	438 (17.0)	181 (20.8)		Reference	<.001					
	25-29		1065 (30.9)	761 (29.6)	304 (34.9)		1.03 (0.83-1.29)	—					
	20-24		1191 (34.6)	883 (34.3)	308 (35.4)		1.18 (0.95-1.47)	—					
	18-19		339 (9.8)	275 (10.7)	64 (7.3)		1.78 (1.29-2.45)	—					
	13-17		230 (6.7)	216 (8.4)	14 (1.6)		6.38 (3.61-11.25)	—					
**Race or gender, n (%)**													
	**Sexual minority men**								<.001				
		White	1370 (39.78)	1042 (40.49)	328 (37.67)		Reference				Reference		
		Latinx	536 (15.56)	400 (15.55)	136 (15.61)		0.93 (0.74-1.17)				1.09 (0.80-1.48)		
		Black	317 (9.2)	188 (7.31)	129 (14.83)		0.46 (0.36-0.59)				0.64 (0.44-0.92)		
		Asian	125 (3.63)	83 (3.23)	42 (4.82)		0.62 (0.42-0.92)				0.58 (0.35-1.97)		
		Other	265 (7.69)	197 (7.66)	68 (7.78)		0.91 (0.67-1.23)				1.09 (0.72-1.63)		
	**Trans or nonbinary**												
		White	443 (12.86)	368 (14.3)	75 (8.56)		1.54 (1.17-2.04)	—			1.00 (0.70-1.43)		
		Latinx	144 (4.18)	115 (4.47)	29 (3.34)		1.25 (0.82-1.91)	—			1.10 (0.63-1.91)		
		Black	100 (2.9)	65 (2.53)	35 (4.02)		0.58 (0.38-0.90)	—			0.53 (0.27-1.04)		
		Asian	29 (0.84)	22 (0.86)	7 (0.78)		0.99 (0.42-2.34)	—			0.42 (0.14-1.26)		
		Other	115 (3.34)	93 (3.61)	22 (2.45)		1.33 (0.82-2.15)	—			0.92 (0.50-1.69)		
**Location^d^, n (%)**													
	**EHE^e^**								<.001				
		Midwest	1124 (32.63)	801 (31.13)		323 (37.07)	Reference				Reference		
		South	449 (13.04)	329 (12.79)		120 (13.78)	1.11 (0.86-1.41)				0.72 (0.52-1.00)		
		Northeast	183 (5.31)	105 (4.08)		78 (9.03)	0.54 (0.39-0.75)				0.47 (0.30-0.71)		
		West	241 (6.99)	156 (6.06)		85 (9.82)	0.74 (0.55-0.99)				0.44 (0.30-0.65)		
	**Non-EHE**												
		Midwest	572 (16.61)	481 (18.69)		91 (10.39)	2.13(1.65-2.76)	—			1.13 (0.81-1.57)		
		South	358 (10.39)	293 (11.39)		65 (7.47)	1.82 (1.35-2.45)	—			1.19 (0.80-1.78)		
		Northeast	291 (8.45)	235 (9.13)		56 (6.42)	1.69 (1.23-2.33)	—			0.96 (0.63-1.45)		
		West	226 (6.56)	173 (6.72)		53 (6.08)	1.32 (0.94-1.84)	—			0.66 (0.43-1.03)		
**HIV status, n (%)**										<.001			—
	HIV negative		2561 (74.36)	2228 (86.59)	333 (38.18)		Reference						
	PrEP^f^ or PEP^g^		769 (22.33)	313 (12.16)	456 (52.36)		0.10 (0.09-0.12)						
	HIV positive		114 (3.31)	32 (1.24)	82 (9.34)		0.06 (0.04-0.09)						
**Number of risk criteria^h^, n (%)**										<.001			
	2 or more		1241 (36.03)	756 (29.38)	485 (55.73)		Reference				Reference		
	Less than 2		2203 (63.97)	1817 (70.62)	386 (44.34)		3.02 (2.58-3.54)				1.35 (1.08-1.69)		
**PrEP indicated^i^, n (%)**										.52			—
	Yes		2972 (86.29)	2226 (86.51)	746 (85.56)		Reference						
	No		472 (13.7)	347 (13.53)	125 (14.36)		0.93 (0.75-1.16)						
**Substance use^j^, n (%)**										.003			
	Yes		533 (15.48)	365 (18.58)	168 (22.78)		Reference				Reference		
	No		2133 (61.93)	1597 (81.36)	536 (76.13)		1.37 (1.11-1.69)				0.77 (0.60-1.00)		

^a^OR: odds ratio.

^b^Results of logistic regression (outcome variable=HIV self-test vs. other HIV verification).

^c^Not available.

^d^Denotes missing data.

^e^EHE: ending the HIV Epidemic priority jurisdictions [15].

^f^PrEP: pre-exposure prophylaxis.

^g^PEP: postexposure prophylaxis.

^h^Risk criteria: (a) inconsistent condom use, (b) HIV-positive partner, and/or (c) bacterial sexually transmitted infection in the past 6 months.

^i^Pre-exposure prophylaxis indication is based on Centers for Disease Control and Prevention’s draft of clinical practice guidelines [16]: HIV negative, recent anal or vaginal sex, and one of the following: inconsistent condom usage, HIV-positive partner, or bacterial sexually transmitted infection in past 6 months.

^j^Substance use: cocaine, meth, or heroin use in past 6 months.

### Adjunct Surveys

Response rates to adjunct survey measures were mixed, with some successful surveys leading to independent publications [12,13]. Over half of the participants in the cohort responded to the COVID-19 survey (2182/3444, 67.26%) [13]. The Truvada lawsuit survey was offered to HIV-negative participants in the cohort, and again, over half (1485/2555, 58.12%) of those eligible participated [12]. The survey of eligible youth who did not enroll had a much lower response rate, with 33 (33/246, 13.4%) of participants completing or partially completing the survey of the 246 participants who were invited to participate. The mean age of participants was 18.6 years (SD 1.5). The majority of participants were cisgender men (21/33, 64%), White (14/33, 42%), gay (13/33, 39%), had completed high school (27/33, 82%), and knew their HIV status (30/33, 91%). When asked about their number one barrier to participate, the most common response was the HIV testing requirement (13/28, 46%), followed by the length of time to receive the test kit and payment (6/28, 21%), the length of the survey (4/28, 14%), and the fact that the study used a website rather than an app (3/28, 11%). HIV testing was particularly a barrier for younger participants, with a majority (9/11, 82%) of younger participants reporting HIV testing as their number one barrier, compared with about one quarter (5/21, 24%) of older participants. When asked about the best platform to advertise the study on, 58% (18/31) of participants reported Instagram to be the best platform, followed by Twitter (5/31, 16%) and Tiktok (3/31, 10%). Most participants agreed (29/30, 97%) that US $20 was an appropriate compensation for a short survey. Many participants expressed some privacy concerns, including the privacy of the HIV testing results (12/29, 41%), privacy in testing, emailing, and receiving mail from staff (11/30, 37%), not being out at home as LGBTQ+ (9/29, 31%), and the privacy of the web-based survey (3/29, 10%).

## Discussion

### Principal Findings

This evaluation of participants enrolled in the Keeping it LITE study indicates that electronic methods are feasible for recruitment of a large, diverse sample of youth and young adults at risk for HIV. We recruited participants representing a broad range of racial, ethnic, gender, and economic groups, although there was limited success in recruiting minor participants. The original goal of recruiting a sample that contained 20% of participants under the age of 18 was not achieved, as the final sample included only 5.95% (205/3444) participants aged <18 years. The racial identity of participants (Table 2) was similar to the 2019 US census estimates [17], although the sample included a slightly smaller proportion of White individuals. Recruitment of transgender and genderqueer participants was an area of success for this study, especially with the large number of trans men recruited, a notably understudied population in HIV research [18,19]. Finally, the study was successful in recruiting participants who were recently diagnosed with HIV, as well as those who were using PrEP and PEP. The rates of PrEP use among HIV-negative participants in this study (769/3330, 23.09%) were comparable with recent estimates of PrEP use compiled by the National HIV Behavioral Surveillance Study [20].

The Keeping it LITE study was 1 of 4 studies funded by the National Institutes of Health to investigate the implementation of large-scale digital HIV interventions for sexual minority men and transgender persons [21-23]. All studies have found success in recruiting large, web-based samples of individuals at risk for HIV, including transgender women [21] and sexual minority men [22]. However, the Keeping it LITE study was able to recruit a larger sample of minor youth compared with other studies recruiting youth [22,23] and was the only study to recruit youth aged 13-15 years. In addition, the only other study that included transgender men [23] recruited a much smaller sample (n=53) than this study (n=277). Thus, although this study did not meet the recruitment goal for minor participants, recruitment of minors and transgender men remains a unique contribution of these methods to the literature.

### YAB Successes

The formation and use of YAB was particularly successful in this study. The success in formation was in part because of having an established YAB at the sponsoring institution. The researchers were able to build on the established structure with additional youth advisors from around the country. In addition, the researchers used the community guidelines and resources established by the National Institute of Allergy and Infectious Diseases–funded HIV/AIDS Network Collaboration and trained staff to be *adult accomplices*, learning to work alongside youth as equal partners. On the basis of this experience, researchers recommend using and building off existing community engagement structures and providing research staff communications training to address health issues among diverse youth.

### Differences Between Recruitment Methods

Most participants were recruited through social media advertisements (1684/2649, 63.57%), although other methods were notably effective for recruiting specific populations. For instance, although word-of-mouth recruitment only accounted for about 18.83% (499/2649) of overall recruitment, this method was vital in the recruitment of Black and trans women participants, leading to the recruitment of nearly half of the trans women and one-third of Black participants. In addition, LGBTQ+ apps only recruited about 12.87% (341/2649) of the overall sample but allowed for the recruitment of individuals with a higher risk profile than other methods. Finally, having a variety of recruitment methods was vital to bolster the diversity of the sample.

### Differences in Enrollment Across Demographics

Demographic disparities in enrollment highlighted groups that may face greater barriers to study participation. There were lower enrollment rates for participants who were genderqueer, minors, persons of color (excluding Asian participants), and not using PrEP or PEP. Of concern, many of these demographic groups, including Black and Hispanic or Latinx individuals and those not using PrEP or PEP, are the groups with the greatest burden of HIV risk. For instance, Black and Latino sexual minority men accounted for 38% and 33% of new HIV diagnoses among sexual minority men in 2019, respectively [24], but of the sexual minority men in this study’s sample, only 13% were Black and 21% were Latinx. Although this study was not able to recruit a sample that is fully representative of those with new HIV infections, the sample did match US racial or ethnic demographics, and included a large number of gender-diverse participants. The sample also contained a representative number of PrEP and PEP users based on recent data on PrEP uptake among sexual minority men [25]. This speaks to the efforts made to be inclusive and widespread in the advertisement for the study.

### Feasibility of At-home Testing

At-home self-testing, a relatively novel outcome assessment method for HIV research trials, proved to be an acceptable, and in fact preferred, method for this sample. The only demographic group in which the majority did not test with an at-home testing kit were PrEP and PEP users. This is likely because they were getting HIV tested as part of their care. In particular, young participants were much more likely to use at-home testing, with nearly all minor participants opting to use this method. Trans men were also more likely to use at-home testing kits, likely because far fewer trans men in this sample used PrEP or PEP.

### Feasibility of Adjunct Surveys

Of the adjunct surveys administered during the course of this study, surveys assessing the impact of the Truvada lawsuit and COVID-19 pandemic proved to be easy to administer and provided useful data quickly on large numbers of participants. The adjunct survey administered to youth who did not enroll was less successful because of limited participation. This was an understandable outcome, given that participants who failed to engage in the study were likely to also be less engaged with a follow-up survey. However, it should be noted that this study design allowed for rapid administration of timely surveys, and participant response, when surveying enrolled participants, was efficient and effective.

### Adaptations to Barriers in Recruitment of Minor Participants

Minor participants proved particularly challenging to recruit for this study because (1) they tended to have fewer HIV risk behaviors and were therefore less likely to qualify, (2) they were less likely to enroll if eligible, and (3) there were difficulties in advertising to minors on some social media platforms (ie, Snapchat). These barriers were addressed by (1) lowering the risk profile required to participate for minors, (2) shifting social media advertising to platforms that allowed advertising to minors and were commonly used by youth (ie, Instagram), and (3) adjusting advertisement content to avoid being flagged as *inappropriate*. Those who responded to the survey of nonparticipant youth indicated that the most common barrier to participating was the HIV testing requirement and the logistics around it (ie, time to receive kit and privacy concerns). The challenges of recruiting minor participants for this study reflected the researchers’ previous experience that younger participants need a larger number of touchpoints in longitudinal studies: more frequent personal interaction, more frequent reminders, and more practical help with study procedures than older participants [26].

### Conclusions

The results of enrollment for the Keeping it LITE study indicate that limited interaction recruitment is feasible and well accepted among youth and young adults at risk for HIV. The study was able to quickly enroll a large cohort that broadly reflects the demographics of HIV infections in the US, though enriched for trans- and gender-nonconforming individuals. Several significant barriers to the limited interaction enrollment of adolescent participants were identified. Although some of these barriers were overcome with creative recruitment methods and input from the YAB, remote enrollment, and particularly at-home HIV testing, are not likely to be successful in enrolling minors at risk for HIV.

Although this cohort study was designed to specifically address HIV incidence and prevention, the framework developed for this study could also be used to investigate social and behavioral epidemiological research questions for sexual and gender minority youth that go beyond HIV. It could also be adapted to use mixed methods, such as incorporating survey questionnaires with open-ended questions, and recruiting a large enough cohort to power inter- and intracategorical intersectional analyses. Regardless of future adaptations, the current implementation demonstrated that consistency is important for retention, such as regular communication with participants, adjusting procedures in response to participant feedback, and updating study incentives to align with participant preferences.

Going forward, this unique cohort will provide invaluable data to inform prevention strategies and to articulate the best methodologies for limited interaction research. We intend to meticulously characterize factors that put youth and young adults at risk for HIV but also describe the personal and environmental characteristics associated with healthy behaviors and successful choices as participants mature. Our aims include thorough characterization of PrEP attitudes, uptake and adherence, and the predictors of successful navigation through the HIV continuum of care for youth.

## Abbreviations

LGBTQ: lesbian, gay, bisexual, transgender, and queer
OR: odds ratio
PEP: postexposure prophylaxis
PrEP: pre-exposure prophylaxis
STI: sexually transmitted infection
YAB: youth advisory board
